# Assessment of increasing intravenous adenosine dose in fractional flow reserve

**DOI:** 10.1186/s12872-016-0463-4

**Published:** 2017-02-14

**Authors:** David Sparv, Matthias Götberg, Jan Harnek, Tobias Persson, Bjarne Madsen Hardig, David Erlinge

**Affiliations:** 1 Department of Cardiology, Clinical Sciences, Lund University, Lund, Sweden; 2 Department of Coronary Heart Disease, Skane University Hospital, Lund, Sweden

**Keywords:** Coronary heart disease, Fractional flow reserve, Adenosine, Visual analogue scale

## Abstract

**Background:**

Effects of increased adenosine dose in the assessment of fractional flow reserve (FFR) were studied in relation to FFR results, hemodynamic effects and patient discomfort. FFR require maximal hyperemia mediated by adenosine. Standard dose is 140 μg/kg/min administrated intravenously. Higher doses are commonly used in clinical practice, but an extensive comparison between standard intravenous dose and a high dose (220 μg/kg/min) has previously not been performed.

**Methods:**

Seventy-five patients undergoing FFR received standard dose adenosine, followed by high dose adenosine. FFR, mean arterial pressure (MAP) and heart rate (HR) were analyzed. Patient discomfort measured by Visual Analogue Scale (VAS) was assessed.

**Results:**

No significant difference was found between the doses in FFR value (0.85 [0.79–0.90] vs 0.85 [0.79–0.89], *p* = 0.24). The two doses correlated well irrespective of lesion severity (*r* = 0.86, slope = 0.89, *p* = <0.001). There were no differences in MAP or HR. Patient discomfort was more pronounced using high dose adenosine (8.0 [5.0–9.0]) versus standard dose (5.0 [2.0–7.0]), *p* = <0.001.

**Conclusions:**

Increased dose adenosine does not improve hyperemia and is associated with increased patient discomfort. Our findings do not support the use of high dose adenosine.

**Trial registration:**

Retrospective Trial registration: Current Controlled Trials ISRCTN14618196. Registered 15 December 2016.

## Background

Fractional Flow Reserve (FFR) is the most validated diagnostic method to determine physiologically significance of stenosis in the epicardial arteries [1–4]. FFR is defined as the ratio of pressure difference across a coronary lesion during hyperemia [5]. The use of FFR-guidance in percutaneous coronary intervention (PCI) has been shown to identify which lesions that benefit from revascularization [2]. In addition, the use of FFR in PCI reduces the need of repeat interventions, enhances quality of life and demonstrates cost effectiveness [3]. FFR carries a strong guideline recommendation (Class I, level of evidence A) [6].

The diagnostic accuracy of FFR in terms of identification of stenosis with inducible ischemia is 85–93% [7–9]. The use of FFR and hence, diagnostic accuracy, requires induction of maximal hyperemia in the coronary arteries [10]. Different agents for induction of hyperemia have been studied, where the most validated are adenosine and papaverine [11, 12]. The purine nucleoside adenosine is a potent vasodilator with short duration time, why dosage in relation to hyperemic effect is unpredictable [13]. In addition, side effects such as hypotension, bradyarrhytmias, respiratory distress and patient discomfort are common [14, 15]. Side effects, together with the cost of adenosine, are considered reasons why the utilization of FFR is still not according to guidelines [16]. Alternatives to adenosine with less side effects of vasodilatation have been suggested, for example the use of non-ionic, radiocontrast medium which generates submaximal hyperemia by osmosis [17, 18]. Also, other techniques such as resting distal pressure (Pd)/aortic pressure (Pa) and the instantaneous wave-free ratio (iFR) are under development [9].

Even though different agents and techniques may offer alternative for future improvements, adenosine, administered by an intravenous infusion of 140 μg/kg/min, remains the golden standard [1, 3, 4, 10, 19].

The adequate dose and administration route of adenosine have been questioned over the years. Previous trials have shown equivalent FFR results by intracoronary injections compared to intravenous infusion [20–23]. Furthermore, optimal dosage for adenosine administration remains unclear and studies have suggested that the current standard dose may be insufficient to induce maximal hyperemia [24–27]. In a previous smaller trial investigating the effects of increased intravenous adenosine, no benefit emerged from the high dose regime [18]. Nevertheless, it is still praxis to increase the hyperemic agent in borderline cases [28]. Considering these discrepancies between trial results and clinical traditions, we wanted to determine if increased intravenous adenosine improve diagnostics in a larger clinical population.

Also, the impact of patient co-morbidity such as chronic kidney disease and diabetes [29], as well as different adenosine antagonists, for example caffeine, has been investigated. In recent trials, caffeine seems to attenuate the effect of adenosine [30, 31]. Caffeine is a derived methylxanthine acting as a competitive inhibitor of adenosine receptors A_1_ and A_2A_. Caffeine consumption prior to adenosine perfusion diagnostics is therefore considered a relative contraindication [32–37].

The primary objective was to study the effects of increased dose intravenous adenosine in FFR. Secondary objectives were to study the hemodynamic effects and patient discomfort of increased adenosine dose in patients with or without caffeine consumption prior to FFR.

## Methods

### Design

The present study was a prospective, non-randomized trial with an open-label design. The non-randomized design was chosen since the patients constitute their own controls. The lower dose was always administered first in order to mimic clinical routine. The study was conducted as a single-center, non-consecutive trial at Skane University Hospital, Lund, Sweden. Patient admitted for coronary angiography were considered eligible for inclusion if the following criteria were fulfilled: Age ≥18 years, borderline-significant coronary stenosis (indication for FFR according to ESC Guidelines) [6] and signed informed consent prior to enrollment. Key exclusion criteria were allergy to adenosine or contrast media, baseline mean arterial pressure <60 mmHg, baseline heart rate <50, pharmacological treated asthma, chronic obstructive pulmonary disease equivalent to GOLD classification III and IV [38], confusion or inability to comprehend the study information.

### Procedure

Following coronary angiography and intracoronary administration of 200 μg Nitroglycerin, a 0.014-inch pressure guide wire (Primewire Prestige®/Verrata® Pressure Guide Wire, Volcano Corporation, San Diego, CA, US) was advanced through a 6-F guide catheter into the coronary artery, calibrated and subsequently advanced distal of the lesion. The infusion of intravenous adenosine (Adenosin Life Medical 5 mg/ml, Life Medical Sweden AB) was started at a weight-adjusted rate, equivalent to standard dose 140 μg/kg/min and terminated when the two minutes measurement was completed. The agent was administrated through a peripheral intravenous line. FFR was recorded for two minutes (±5 s) and calculated by the Volcano CORE™ integrated system with the S5I® software and Case Manager (Volcano Corporation, San Diego, CA, US). Prior to the second measurement, a recovery time was mandatory for the pressure curve to return to baseline values (minimum 5 min). After recovery, the second measurement was performed with similar FFR technique and an intravenous adenosine infusion of 220 μg/kg/min. FFR was considered significant if ≤0.80. The FFR results of standard dose were used for clinical decision of revascularization. A ≥0.02 drift of the FFR-wire was considered clinical relevant, and if this occured, a new calibration was performed. Consumption of caffeine was defined as a minimum of 200 ml filter coffee consumed ≤6 h prior to FFR. The patients coffee intake ranged between 200 and 400 ml.

### Data collection

Mean arterial pressure (MAP) and heart rate were monitored and collected at baseline and every 30 s during the measurements (Philips Intellivue® Cardiac system, version M8010A, Royal Philips Electronics, Amsterdam, Netherlands). The patients were asked to score maximum discomfort following each measurement by a validated tool for determination of pain/discomfort, the Visual-Analogue-Scale (VAS) [39–41].

### Sample size and statistical analysis

Based on standard deviations in FFR-measurements in previous studies [24, 25], a sample size of 72 would have a 90% power to detect a 15% difference of FFR with a significance level (alpha) of 0.05% (two-tailed). In order to compensate for loss of data due to a presumed inability to tolerate high dose adenosine, the a priori number of patients intended to include was 85. The statistical analysis was performed using the Graphpad Prism6™ for Mac OS X™ (Graphpad Software Inc. La Jolla, CA, US). Continuous variables were followed and reported as a mean ± standard deviation or as median (interquartile range 25–75) if asymmetrically distributed. The correlation of adenosine doses was calculated by Wilcoxon matched-pairs signed rank test and a linear regression model. The agreement was graphically displayed in a Bland-Altman plot. In addition, for the continuous hemodynamic variables of mean arterial pressure and heart rate, an area under curve (AUC) analysis was performed. The interference between the groups in the caffeine analyses was based on the computation of the Mann-Whitney U-test (Wilcoxon rank-sum-test) to determine significance. A *p*-value of <0.05 was considered statistically significant.

## Results

### Patient characteristics and procedural data

Eighty-seven patients scheduled for FFR were included. Ten patients (11%) developed atrioventricular block during administration of standard dose adenosine and were excluded from the second measurement and further analysis. Another 2 patients declined to participate in the second measurement due to severe discomfort from adenosine administration. Of the remaining 75 patients, two complete FFR measurements within the same coronary lesion using the two different dose regimens were performed. Patient demographics and clinical characteristic are presented in Table 1. In 28% of the cases, FFR was ≤0.80 after the first measurement and PCI were thus performed. In the group treated by PCI, procedural success was 100%. Stable angina (Canadian Class I-II) was the most common indication for FFR (36%). In the group of acute coronary syndrome, the non-culprit vessel was used for the current trial. In 49.3% of the cases, LAD was the target FFR vessel and in 41.3%, the lesion location was proximal (Table 2.) 89.5% of the patients were treated by dual antiplatelet therapy (Table 3).

**Table 1:** Baseline characteristics and indiciations for angiography

Patient demographics and clinical characteristics	
Male (%)	77.3
Age (yrs)	66 ± 10
Body mass index (kg/m2)	26 ± 4
S-Creatinine (μmol/l)	87 ± 34
Previous MI (%)	33.3
Previous PCI (%)	43.2
Previous CABG (%)	11.8
Diabetes Mellitus (%)	20.2
Current Smoker (%)	14.5
Previous Smoker (%)	46.7
Hypertension (%)	76.0
Hyperlipidemia (%)	64.0
Caffeine consumption prior to procedure (%)	57.3
Indications for coronary angiography	
Stable angina (%)	36.0
Unstable angina (%)	34.7
NSTEMI (%)	28.0
Diagnostic procedures (%)	1.7

Values are percentage and mean ± SD. *MI* myocardial infarction, *PCI* percutaneous coronary intervention, *CABG* coronary artery bypass grafting, *NSTEMI* non ST-elevation myocardial infarction

**Table 2:** Procedural characteristics

Coronary Angiography findings	
Normal findings/Atheromatosis (%)	33.3
1-vessel disease (%)	29.4
2-vessel disease (%)	24.3
3-vessel disease (%)	13.0
Target vessel FFR	
Left Main (%)	2.7
LAD (%)	49.3
1st Diagnonal branch (%)	4.0
LCx (%)	10.7
1st Marginal branch (%)	10.7
PLA (%)	2.6
RCA n (%)	20
Lesion classification	
Type A	37.3
Type B1	26.7
Type B2	14.7
Type C	20.3
Lesion characteristics	
Proximal	41.3
Mid	40.7
Distal	17
Bifurcation	9.3
In-stent restenosis	2.7

Values are presented as %. *LAD* left anterior descending coronary artery, *LCx* left circumflex coronary artery, *PLA* posterior-lateral artery, *RCA* right coronary artery

**Table 3:** Pharmaceutical therapy

Aspirin (%)	100
Clopidogrel (%)	10.7
Ticagrelor (%)	78.8
Bivalirudin (%)	9.4
Heparin (%)	100
Warfarin (%)	2.5

### FFR measurements

**Fig. 1:**
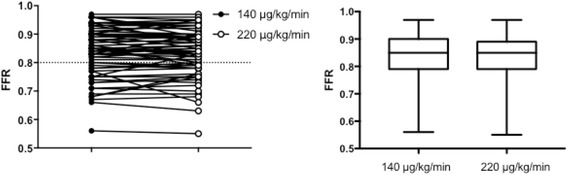
Wilcoxon matched-pairs signed rank test: There was no significant difference in the matched-pairs comparision of intravenous adenosine infusion of 140 μg/kg/min versus 220 μg/kg/min (0.85 [0.79–0.90] vs 0.85 [0.79–0.89], *p* = 0.24)

**Fig. 2:**
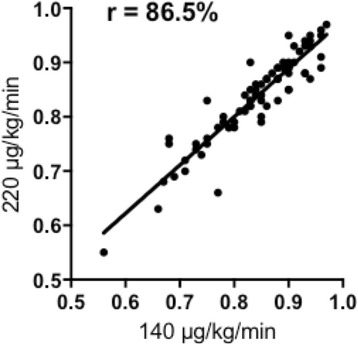
Linear regression model: High dose adenosine showed a strong significant linear correlation to standard dose (*r* = 0.86, slope = 0.89, *p* = <0.001)

**Fig. 3:**
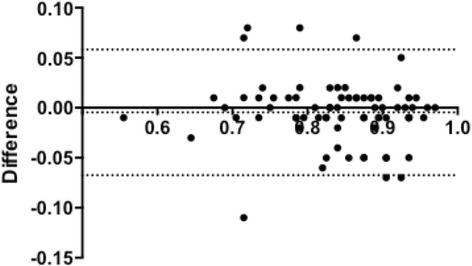
Bland-Altman plot: In the Bland-Altman analysis, average of the differences was -0.005 ± 0.03 (mean bias ± SD) [−0.07 to 0.06], [95% CI]

There was no significant difference in the matched-pairs comparison of intravenous adenosine infusion of 140 μg/kg/min versus 220 μg/kg/min (0.85 [0.79–0.90] vs 0.85 [0.79–0.89], *p* = 0.24) (Fig. 1). In addition, high dose adenosine showed a strong significant linear correlation to standard dose (*r* = 0.86, slope = 0.89, *p* = <0.001) (Fig. 2). In the Bland-Altman analysis, average of the differences were -0.005 ± 0.03 (mean bias ± SD) [-0.07 to 0.06], [95% CI], (Fig. 3). In four patients (5.3%), the higher dose of Adenosine caused a change in agreement due to lowering FFR below the treatment threshold of 0.80 (0.85–0.79, 0.81–0.78, 0.81–0.79 and 0.81–0.79). The high dose did not decrease FFR below 0.75 in any of the 75 cases. Thus, all changes remained in the borderline region.

### Hemodynamic effects and patient discomfort

**Fig. 4:**
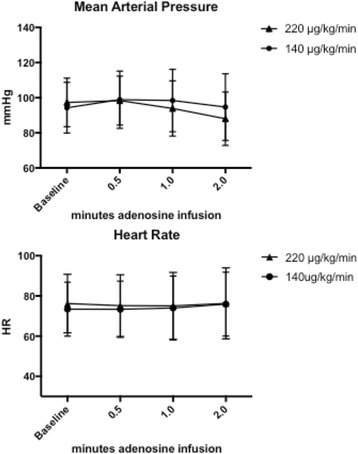
Mean arterial pressure and heart rate. There were no differences in mean arterial pressure or in heart rate for standard dose versus high dose

**Fig. 5:**
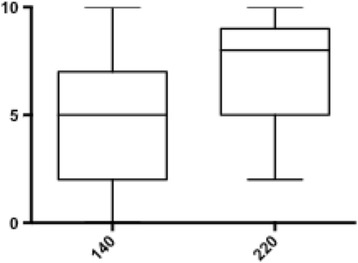
VAS: Patient maximal discomfort during adenosine administration was significantly higher in the dosage of 220 μg/kg/min (8.0 [5.0–9.0]) versus standard dose (5.0 [2.0–7.0]), *p* = <0.001

Mean arterial pressure and heart rate were similar during infusion of the different adenosine doses. MAP: Standard dose 7152 ± 178.2 versus high dose 6991 ± 346.7 AUC [arbitrary units], *p* = 0.34). Heart rate: Standard dose 5488 ± 95.45 versus high dose 5602 ± 49.10 AUC [arbitrary units] *p* = 0.11 (Fig. 4). Ten patients were excluded from the second FFR measurement due to atrioventricular block. In the remaining 75 patients, the occurrence of atrioventricular block and bradyarrhytmias was 5.3%. Patient maximal discomfort during adenosine administration, measured by VAS, was significantly higher in the dosage of 220 μg/kg/min (8.0 [5.0–9.0]) versus standard dose (5.0 [2.0–7.0]), *p* = <0.001 (Fig. 5).

### Caffeine

**Fig. 6:**
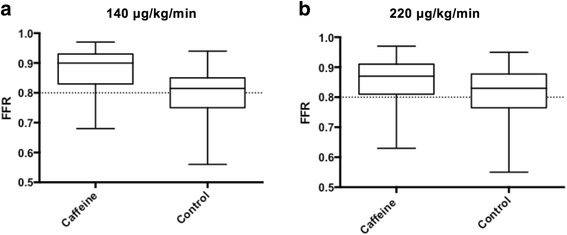
**a** FFR were significantly higher in the caffeine group compared to control (*p* = <0.001). **b** In the high dose regime, there was a similar trend but not significant

**Fig. 7:**
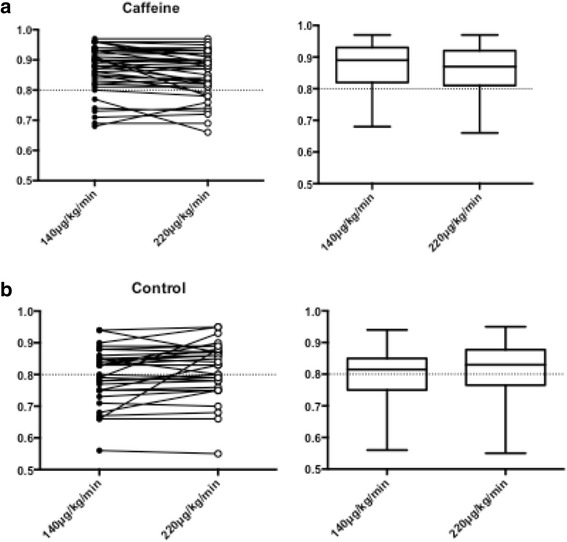
**a** In a paired comparision of caffeine consumption, FFR was significantly higher in the group receiving standard dose versus high dose (0.89 [0.83–0.93 vs 0.87 [0.81–0.91], *p* = <0.001). **b** In the control group, this difference was reversed to significantly lower FFR in standard dose compared to high dose (0.82 [0.75–0.85] vs 0.83 [0.77–0.89], *p* = 0.02)

A subgroup analysis was performed in the 43 patients (57%) of the study population who reported caffeine consumption ≤6h prior to FFR. In four patients (5.3%), three from the caffeine group (4%) and one from the control group (1.3%), high dose adenosine decreased FFR from non-significant to borderline significant (0.78–0.79). In the adenosine dosage of 140 μg/kg/min, FFR was significantly higher in the caffeine group compared to control (0.90 [0.83–0.93] versus 0.82 [0.75–0.85], *p* = <0.001. In the high dose regime, there was a similar trend but not significant (0.87 [0.81-0.91 versus 0.83 [0.77–0.88], *p* = 0.09) (Fig. 6). In a paired comparison of caffeine consumers in the study population, FFR was significantly higher in the group receiving standard dose versus high dose adenosine (0.89 [0.83–0.93 vs 0.87 [0.81–0.91], *p* = <0.001). In the control group, this difference was reversed to significantly lower FFR in standard dose compared to high dose (0.82 [0.75–0.85] vs 0.83 [0.77–0.89], *p* = 0.02) (Fig. 7). In three patients (4%), the higher dose of adenosine caused a change in agreement due to lowering FFR below the treatment threshold of 0.80 (0.85–0.79, 0.81–0.78, 0.81–0.79. The high dose did not decrease FFR below 0.75 in any of the 75 cases.

## Discussion

In this clinical trial we evaluated whether increased dose adenosine in FFR would be associated with improved accuracy of FFR compared to standard dose. No significant differences in FFR values were found. Patient discomfort was significant during administration of the higher dose.

### FFR measurements

Following the landmark trials FAME [4] and FAME2 [3] the perception of assessment in non-critical coronary stenosis have been altered. FFR became a powerful tool not only for clinical practice, but also as the baseline standard in clinical research [42, 43]. The critical prerequisite of hyperemia and the side effects following systemic response of intravenous administration, have urged development of different agents, routes and doses. Even though different intravenous adenosine doses during FFR been investigated in an earlier smaller study [18], this present study concludes that their results indeed are reproducible in a larger, clinical patient population. Our results demonstrate that an increased dose adenosine do not improve accuracy in FFR, but is associated with increased side effects. Different pharmaceutical agents such as contrast media constitute an interesting option without the side effects of vasodilatation. The hyperemic capacity, mediated by osmosis, of contrast is well known, but has in previous trials been inferior to adenosine and papaverin [18]. Recent trials have demonstrated non-inferiority between contrast media and adenosine in the assessment of intermediate stenosis [17, 44], but even though this may be an option in certain lesions, there will still be need of adenosine-induced hyperemia in some cases; hence the question of increased adenosine doses needs to be adressed.

### Hemodynamic effects and patient discomfort

Adenosine interaction with A_2A_ receptor mediates a smooth muscle relaxation and thus a vasodilatation [45]. In addition, adenosine regulates the autonomic innervation in the heart by inhibition of the A_1_ receptors, and by that, accomplishes a negative chronotropic and dromotropic effect in the conduction system [46, 47]. A concern in administration of adenosine, foremost in high dose regime, is that patients will develop adenosine induced systemic hypotension and bradyarrhytmias. In a recent trial, the predictor of hypotension during hyperemia was obesity and decreased microcirculatory resistance [48]. Of the original enrollment of 87 patients, a total of 11.3% developed an atrioventricular block during standard dose administration, and another 5.3% during high dose. Even though bradycardia usually is not persistent, it may create discomfort, which is an important reason why adenosine is prematurely terminated during FFR and hence, the hyperemic effect remains uncertain. Hypotension was uncommon in our study. Hypothetically, patients with unstable angina/NSTEMI and more complex lesions might be more prone to develop hypotension. Also, we cannot rule out that the intermittent monitoring of mean arterial pressure could affect the hemodynamic measurements by terminating infusions before severe hypotension develops. However, the vasodilation capacity of adenosine, displayed by shortness of breath, flushing and severe discomfort, was prominent. This observation confirms that symptoms during adenosine administration are not solely related to a systemic response or coronary hemodynamic changes, but rather via a direct effect on C-fibers in the lungs [49].

### Caffeine

In this study, caffeine consumption was not randomized or controlled, but in a secondary analysis we found trends coherent with recent findings [31]. Matsumoto et al. [30] investigated the effect of intravenous adenosine doses of 140, 175 and 210 μg/kg/min in relation to papaverine during FFR. The effect of caffeine was less prominent at 220 μg/kg/min. The increased FFR results in patients on caffeine compared to controls in our trial may indicate an attenuated adenosine effect by coffee. However, the effect of increasing the dose of adenosine was marginal.

### Limitations of the study

The present study has some limitations. The higher dose was always administered after the lower dose and a cross-over design might have yielded different results. However, this is the way adenosine is used in clinical practice, and no one would consider starting with the higher dose in all patients. The dose and timing of caffeine consumption were neither randomized nor controlled which might generate confounders. This was, however, an exploratory secondary endpoint. In terms of adenosine administration, we used a peripheral intravenous line, which compared to a central vein might have a slightly delayed systemic effect. However, we did flush the adenosine infusion together with an infusion of saline, a validated method to increase bioavailability and making the possible delay negligible. Also, in the era of transradial approach, a peripheral route for adenosin is desirable, and has been demostrating similar results in recent findings [50].

A considerable number of stable patients with type A lesions were enrolled. Although this reflects standard use of FFR in a real-life patient cohort, the possibility that a population with more advanced disease would have different results, cannot be ruled out.

## Conclusion

Increased dose adenosine in fractional flow reserve measurements did not lower the FFR but was associated with a significant increase of patient discomfort. We did not observe persistent adenosine-induced hypotension and the occurrence of bradyarrhytmias were similar between standard dose and high dose. Our findings do not support high dose adenosine in the assessment of fractional flow reserve.

## Abbreviations

AUC: Area under curve
ESC: European Society of Cardiology
FFR: Fractional flow reserve
GOLD: Global initiative for chronic obstructive lung disease
HR: Heart rate
LAD: Left anterior descending artery
MAP: Mean arterial pressure (mmHg)
PCI: Percutaneous coronary intervention
VAS: Visual-analogue-scale
